# CNS Recruitment of CD8+ T Lymphocytes Specific for a Peripheral Virus Infection Triggers Neuropathogenesis during Polymicrobial Challenge

**DOI:** 10.1371/journal.ppat.1002462

**Published:** 2011-12-22

**Authors:** Christine M. Matullo, Kevin J. O'Regan, Mark Curtis, Glenn F. Rall

**Affiliations:** 1 Fox Chase Cancer Center, Division of Basic Science, Program in Immune Cell Development and Host Defense, Philadelphia, Pennsylvania, United States of America; 2 Thomas Jefferson University, Department of Microbiology and Immunology, Philadelphia, Pennsylvania, United States of America; 3 Thomas Jefferson University, Department of Pathology, Anatomy, and Cell Biology, Philadelphia, Pennsylvania, United States of America; University of North Carolina at Chapel Hill, United States of America

## Abstract

Although viruses have been implicated in central nervous system (CNS) diseases of unknown etiology, including multiple sclerosis and amyotrophic lateral sclerosis, the reproducible identification of viral triggers in such diseases has been largely unsuccessful. Here, we explore the hypothesis that viruses need not replicate in the tissue in which they cause disease; specifically, that a peripheral infection might trigger CNS pathology. To test this idea, we utilized a transgenic mouse model in which we found that immune cells responding to a peripheral infection are recruited to the CNS, where they trigger neurological damage. In this model, mice are infected with both CNS-restricted measles virus (MV) and peripherally restricted lymphocytic choriomeningitis virus (LCMV). While infection with either virus alone resulted in no illness, infection with both viruses caused disease in all mice, with ∼50% dying following seizures. Co-infection resulted in a 12-fold increase in the number of CD8^+^ T cells in the brain as compared to MV infection alone. Tetramer analysis revealed that a substantial proportion (>35%) of these infiltrating CD8^+^ lymphocytes were LCMV-specific, despite no detectable LCMV in CNS tissues. Mechanistically, CNS disease was due to edema, induced in a CD8-dependent but perforin-independent manner, and brain herniation, similar to that observed in mice challenged intracerebrally with LCMV. These results indicate that T cell trafficking can be influenced by other ongoing immune challenges, and that CD8^+^ T cell recruitment to the brain can trigger CNS disease in the apparent absence of cognate antigen. By extrapolation, human CNS diseases of unknown etiology need not be associated with infection with any particular agent; rather, a condition that compromises and activates the blood-brain barrier and adjacent brain parenchyma can render the CNS susceptible to pathogen-independent immune attack.

## Author Summary

There are many CNS diseases, including multiple sclerosis and amyotrophic lateral sclerosis, which have an inflammatory component, though no direct link has been established between incidence and a CNS-resident infectious agent. We reasoned that peripheral immunogens could play a role in CNS disease by inducing an immune response that is “mis-targeted” to the brain. This hypothesis was based on the immunological principle that, while education and activation of naïve cells is an antigen-driven process, recruitment is primarily antigen-independent. We developed a viral co-infection model using measles virus (MV) as a CNS activator and recruiting signal and lymphocytic choriomeningitis (LCMV) as a peripheral immune response initiator. Co-infection with both viruses resulted in significant morbidity and mortality, coincident with LCMV-specific CD8^+^ T cell trafficking to the brain. Death occurred due to edema, despite an apparent absence of LCMV antigens within the brain, and pathogenesis was CD8^+^ T cell-dependent, but perforin-independent. Thus, recruitment of peripherally activated CD8^+^ T cells to the CNS can potentiate neuroinflammation. This work raises the possibility that concomitant immune challenges may be an important cause of the neuroinflammation of some human CNS diseases, perhaps accounting for the inability to identify a discrete pathogenic trigger within affected brain tissues.

## Introduction

Despite the exquisitely specific activation of the adaptive immune response following antigenic encounter, recruitment of immune cells to the affected site is governed by relatively nonspecific factors, including chemokine gradients and adhesion molecule induction on barrier endothelia [1]–[3]. Indeed, some studies have shown that activated immune cells can be recruited to a tissue where no cognate antigen exists. For example, using a mouse model of influenza infection, it was shown that primed transgenic CD4^+^ T cells that were specific for ovalbumin (with no cross-reactivity to flu) migrated efficiently to the infected lung. Despite such recruitment, these cells did not proliferate [4], showing that T cell recruitment and proliferation can be uncoupled.

The complexity of concurrent immune challenges that humans are likely to encounter is staggering, including myriad combinations of pathogens, allergens, and vaccines. In fact, many human and animal diseases are caused by polymicrobial exposures, including human pneumonia, otitis media, peritonitis and periodontitis. Other diseases, such as hepatitis and Lyme's disease, though caused by a single pathogen, can have exacerbated symptoms when combined with a second pathogen [5], [6]. In light of the observed antigen-independent recruitment of activated immune cells, an understanding of the trafficking and function of immune cells beyond the traditional “single pathogen challenge” approach that most viral pathogenesis studies employ is paramount. Specifically in this report, we asked whether recruitment of activated immune cells to virus-negative tissues occurs in individuals who are challenged simultaneously with multiple pathogens/antigens of differing tropism, and if so, whether this affects the pathogenic outcome.

The studies reported here focus on the consequences of immune cell recruitment into the CNS, as the unique environment of the brain (e.g., restricted opportunity for inflammation, nonrenewable cell populations) may make this organ system particularly vulnerable. Moreover, given the number of CNS diseases of unknown etiology that have an inflammatory component, this work may be relevant to future translational efforts to deduce the origins of such conditions.

As a model, we infected permissive mice with neuron-restricted measles virus (MV) and peripherally-restricted lymphocytic choriomeningitis virus (LCMV). Measles is a member of the family *Paramyxoviridae*; natural infection is restricted to humans and primates. The identification of CD46 and SLAM as human MV receptors allowed for the establishment of transgenic mice that express these receptors in specific tissues. In NSE-CD46^+^ mice, CD46 receptor transcription is restricted to CNS neurons by control of the neuron-specific enolase (NSE) promoter [7]. Neuronal infection occurs in all NSE-CD46^+^ mice [8], though CNS disease occurs only in neonates and immunocompromised adults. Adult immunocompetent mice clear the infection without pathogenic consequences, chiefly via interferon gamma (IFN-γ) [9].

Lymphocytic choriomeningitis virus is a member of the family *Arenaviridae*, a natural pathogen of mice, and a historically important tool in uncovering basic aspects of viral immunity [10], [11]. One of the notable attributes of LCMV is the diversity of consequences that can be achieved in infected mice by varying viral parameters (including the route of inoculation, dose, and viral strain), as well as host features (including mouse strain, age, and immunocompetence). Two of these outcomes are relevant here. Following intraperitoneal (IP) inoculation of adult, immunocompetent mice, LCMV establishes infection of peripheral organs including the spleen, liver, and kidney. By 4-6 days post-infection (dpi), the host mounts a robust T cell response (principally CD8^+^ T cells, with 80–95% of the splenocytes being specific for LCMV [12]) that resolves the infection within 7–10 days in the absence of overt disease. In contrast, delivery of as few as 1 plaque forming unit (PFU) of LCMV into immunocompetent adults by an intracerebral (IC) route results in lethal choriomeningitis within 6–7 dpi [13]. By this route, LCMV rapidly establishes infection of the membranes of the brain, including the meninges, leptomeninges, and ependyma, as well as the cerebro-spinal fluid (CSF)-producing choroid plexus cells within the ventricles. As with the peripheral infection, virus-specific CD8^+^ T cells rapidly expand and migrate to the infected tissues. In the case of an LCMV IC infection, these cells migrate to the CNS, where they then cause damage to the ventricular membranes, resulting in edema and precipitous uncal herniation [14].

In this study, we have taken advantage of the unique tissue tropism of transgene-restricted neuronal infection with MV and route of inoculation-restricted peripheral infection with LCMV to evaluate the pathogenic consequences following the recruitment of activated, peripheral LCMV-specific lymphocytes to the CNS, despite a lack of LCMV infection in this tissue. We show that upon concomitant infection with these two viruses, activated immune cells specific for LCMV are recruited to a “primed” CNS where they trigger neurological disease in the absence of LCMV infection.

## Materials and Methods

### Ethics statement

This study was carried out in accordance with the recommendations in the Guide for the Care and Use of Laboratory Animals of the National Institutes of Health. The protocol was reviewed and approved by the Fox Chase Cancer Center Institutional Animal Care and Use Committee (Office of Laboratory Animal Welfare assurance number: A3285-01).

### Mice, virus and infections

Inbred NSE-CD46^+^, NSE-CD46^+^ mice on a recombinase-activating gene-2 deficient background (RAG-2 KO) [15], and NSE-CD46^+^ mice on a perforin deficient background (PFN KO)[16], were maintained in the closed breeding colony at Fox Chase. All animals were on the H-2^b^ background. LCMV Armstrong (LCMV-Arm; ATCC) was passaged in BHK-21 fibroblasts and plaque purified, and titers were determined on Vero fibroblasts. MV-Edmonston (MV-Ed; ATCC) was also passaged and titered on Vero cells.

Mice were infected IC along the midline with the indicated PFU of LCMV-Arm, MV-Ed, or phosphate buffered saline (PBS) as a control, in a total volume of 15–20 µl using a sterile 27-gauge needle. LCMV was delivered by an IP route in a volume of 200 µl using a sterile 25-gauge needle. All mice were anesthetized with metofane prior to inoculation and were monitored daily for signs of illness. For some experiments, weight change was determined by establishing the pre-infection baseline and subsequently calculating weight gain or loss. Moribund mice that lost >20% of their original body weight were euthanized. For some experiments, virus was inactivated by exposure to UV for 15 m; inactivation was verified by subsequent plaque assay. For CD8 depletion experiments, 150 µg of CD8 depleting antibody (purified at the FCCC hybridoma facility from hybridoma clone 2.43 [ATCC #TIB 210]) was injected IP 1d prior to infection and every 7d thereafter throughout the course of study.

### Quantitative RNA analysis

To isolate RNA from infected tissues, brains were snap-frozen in liquid nitrogen and homogenized in TriReagent (Sigma). Thereafter, RNA was purified and quality-tested by gel electrophoresis. Contaminating DNA was removed using TURBO DNA-*free* (Ambion, Austin, TX). RNA was quantified using the Agilent 2100 BioAnalyzer in combination with a RNA 6000 Nano LabChip. RNA was reverse-transcribed using M-MLV reverse transcriptase (Ambion) and a mixture of anchored oligo-dT and random decamers. For each sample, 2 RT reactions were performed with inputs of 100 and 20 ng. An aliquot of the cDNA was used for 5′-nuclease assays using Taqman chemistry. LCMV specific primers, in combination with Universal Master mix, were run on a 7900 HT sequence detection system (Applied Biosystems, Foster City, CA). Cycling conditions were 95°C, 15m followed by 40 (2-step) cycles (95°C, 15s; 60°C, 60s). Relative quantification to the control was done using the comparative C_t_ method. The values plotted are the average of at least 2 PCR reactions, and were normalized to actin.

Measles nucleoprotein: fwd -- CGCAGGACAGTCGAAGGTC,

rev -- TTCCGAGATTCCTGCCATG,

probe -- 6fam-TGACGCCCTGCTTAGGCTGCAA-bhq1.

LCMV nucleoprotein: fwd -- CTAACTATGGCTTGTATGGCCAAA,

rev -- TAAAGCAAGCCAAGGTCTGTGA,

probe -- 6fam-CACAGACTCCGCTCAATGACGTTGTACA-bhq1.

Actb: fwd -- CCAGCAGATGTGGATCAGCA,

rev -- CTTGCGGTGCACGATGG,

probe -- 6fam-CAGGAGTACGATGAGTCCGGCCCC-bhq1.

### Histology and immunohistochemical analysis of mouse tissues

For each timepoint, brains from 4–5 mice were removed and either immersed in 10% formalin for paraffin embedding and subsequent sectioning and staining with hemotoxylin/eosin, or immersed in tissue embedding compound, snap-frozen in a dry ice/isopentane bath and stored at −80°C for immunohistochemistry. Horizontal cryosections (10 µm) were air dried and stored at −80°C. On the day of staining, sections were fixed in ice-cold 95% ethanol, rehydrated in PBS, and blocked for 20 m with 0.1% BSA/PBS, followed by an avidin and biotin block (Vector Laboratories, Burlingame, CA). Rat anti-mouse CD4 (clone RM4-5; 1∶100; Pharmingen, San Diego, CA), or rat anti-mouse CD8a/CD8b.2 antibodies (clones 53-6.7 and 53-5.8, respectively; 1∶100 each; Pharmingen) were used to identify CD4^+^ and CD8^+^ T lymphocytes. Sections were then incubated for 1 h at room temperature with a biotinylated anti-rat IgG secondary antibody at a 1∶200 dilution (Vector). Sections labeled with biotinylated antibodies were treated for 30 m with a streptavidin-peroxidase conjugate (ABC Elite; Vector), followed by visualization with diaminobenzidine (DAB; 0.7 mg/ml in 60 mM Tris) and urea H_2_O_2_ (0.2 mg/ml), purchased as pre-weighed tablets (Sigma). All cells were counterstained with hematoxylin and preserved with an aqueous mounting medium. Uninfected tissues, use of a haplotype matched primary antibody, or omission of the primary antibody served as negative controls. For all histological analyses, at least 3 sections per brain were examined from 3 different horizontal levels, and at least 4 mice per experimental group were assessed.

### Flow cytometric analysis of brain infiltrates

On the indicated dpi, mice were deeply anesthetized with 400 µl 3.8% chloral hydrate in PBS, delivered IP. Once animals were confirmed to be nonresponsive, the mice were perfused with 30 ml PBS. Following perfusion, each brain and spleen was removed and pressed through a nylon mesh cell strainer in PBS. Dissociated tissue was run over a 30/70% discontinuous Percoll gradient for 20 m at 4°C. Mononuclear cells (MNCs) were recovered from the interface, washed with PBS, treated with 0.84% ammonium chloride to remove contaminating red blood cells (RBCs) and washed again. Collected MNCs were counted using a standard hemocytometer and plated into a v-bottom 96-well plate for subsequent antibody staining for multicolor flow cytometry. The following antibodies (eBioscience) were used: PE-Cy5-CD8a, APC-Ax750-CD4, PE-CD3e, FITC-CD11c, APC-CD161c (NK1.1), PB-CD19, PE-Cy5.5 Gr-1 (Ly-6G), PE-Cy7-CD49b (DX5), Ax700-CD11b, APC-NP396 and APC-GP33 tetramers (a generous gift from John Wherry, Univ. Pennsylvania). Cells were allowed to incubate with antibody for 1 h at 4°C and then washed following the incubation period. Pelleted, stained cells were resuspended and read in a BD LSR II system. Percentages obtained from flow cytometry were combined with hemacytometer counts in order to calculate total cell numbers.

### Cytotoxic T lymphocyte (CTL) reaction

CD46-MC57 or MC57 cells were infected, respectively, with either MV or LCMV at a multiplicity of infection (MOI) of 1 for 1 h, or left uninfected, and further cultured for 2d. 3×10^6^ of these target cells were then labeled with 35 µl ^51^Cr for 1 h at 37°C. Single cell suspensions of splenocytes were isolated from mice infected with LCMV IP 6d previously and incubated with ^51^Cr labeled target cells for 6 h at 37°C. Effector and targets cell concentrations were calculated and cells were added at ratios of 100∶1, 50∶1, and 10∶1 in a total reaction volume of 200 µl, with each condition in triplicate. DMEM and 1% IGEPAL (Sigma) were used in place of splenocytes for calculating spontaneous release and maximum release, respectively. Following effector/target cell co-culture, 40 µl of collected supernatant for each condition was diluted in 60 µl MicroScint scintillation fluid (Perkin Elmer). Released ^51^Cr was measured in a gamma counter with the percentage of specific ^51^Cr release being calculated by the following formula: [(experimental release – spontaneous release) / (maximum release – spontaneous release)] x 100.

### Blood-brain barrier permeability and edema measurements

At 7d post-inoculation with LCMV-Arm IP, MV-Ed IC or PBS, mice were deeply anesthetized with 400 µl 3.8% chloral hydrate in PBS, delivered IP. Once animals were confirmed to be nonresponsive, 200 µl of a 2% Evan's Blue solution was then administered transcardially; 2 m post-injection, the mice were perfused with 30 ml PBS. For Evan's blue quantification, brains were dissected, placed in test tubes, snap-frozen in liquid nitrogen and stored at −80°C. Brain tissues were slowly thawed at RT, homogenized in 1 ml N,N-dimethyl formamide and incubated at 50°C for 2d. Following centrifugation at 2700 g for 10 m, the absorbance of the supernatant containing extracted Evan's blue was measured in a spectrophotometer at 620 nm. Each absorbance reading was normalized to wet brain weight. Evan's Blue uptake into the tissues of each infected animal was divided by the average uptake detected in similar tissues from PBS control mice and the results expressed as fold induction. Statistical significance was determined using the Wilcoxon signed-rank test.

To determine brain water weight, mice were euthanized with isofluorane at 7d post-inoculation with LCMV-Arm IP, MV-Ed IC or PBS. For CD8^+^ T cell depletion experiments, 150 µg of CD8 depleting antibody (purified at the FCCC hybridoma facility from hybridoma clone 2.43 [ATCC #TIB 210]) was injected IP 1d prior to infection. Brains were removed, placed in test tubes and then desiccated in a vacuum oven for 24 h at ∼80°C at 15 mm Hg. Brain weights were determined both prior to and immediately following desiccation, with relative percent water weight determined by the following formula: [(wet weight - dry weight)/wet weight] x 100. Water weight of the brain of each infected animal was divided by the average water weight detected in similar tissues from PBS control mice, and the results expressed as percentage change. Statistical significance was determined using the Wilcoxon signed-rank test.

## Results

### Co-infection with neurotropic and viscerotropic viruses leads to unique neuropathogenesis

**Figure 1:**
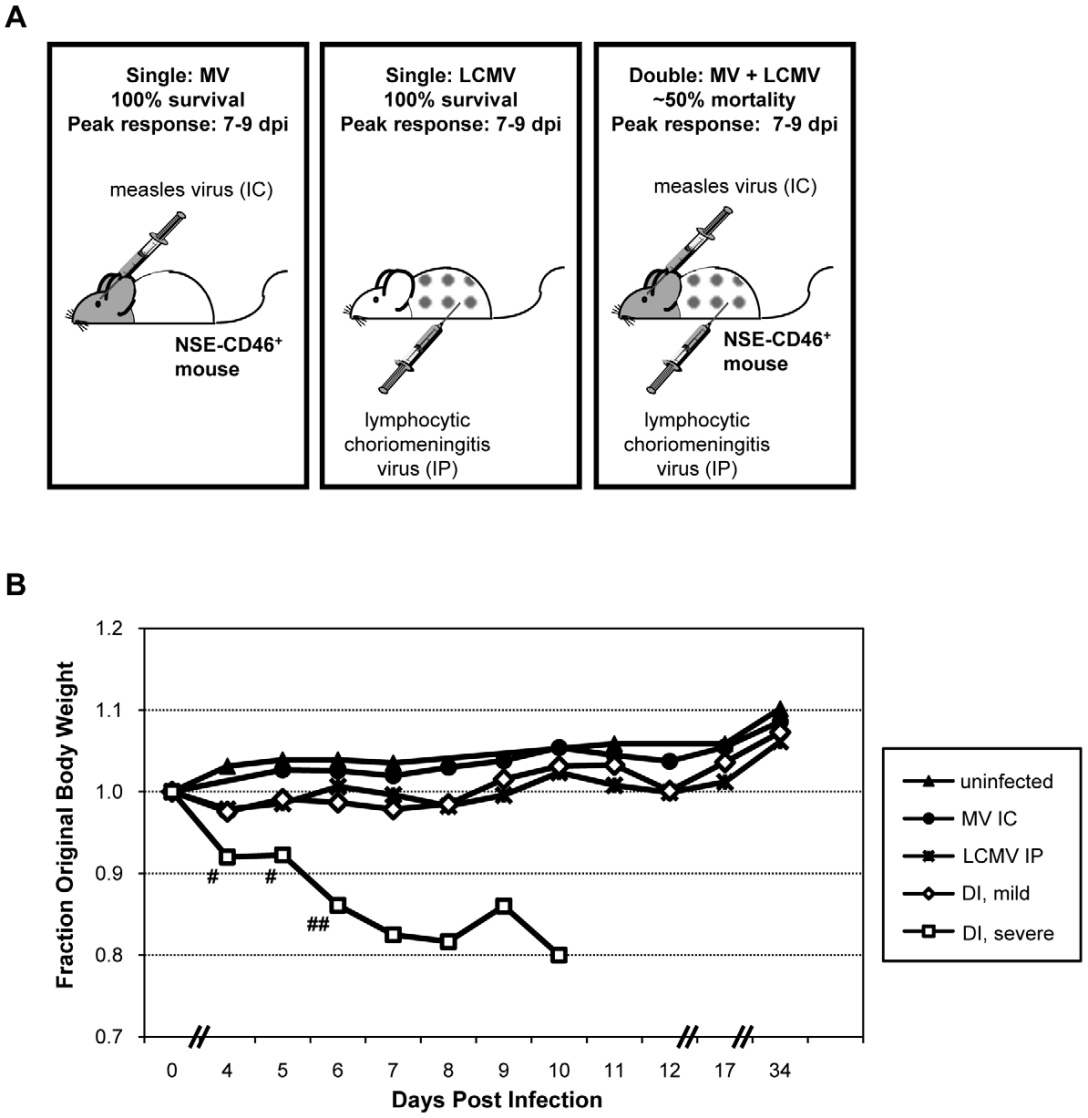
Co-infection model and pathogenic outcome. (A) NSE-CD46^+^ transgenic mice survive MV IC challenge and mount an immune response that peaks between 7–9 dpi (left panel) [9]. LCMV, a natural mouse pathogen, is restricted to peripheral organs (spleen, liver, kidney, pancreas) following IP inoculation (middle panel); all mice survive, and peak immunity also occurs between 7–9 dpi [12]. In contrast, ∼50% of mice infected with both MV IC and LCMV IP (doubly infected; DI) succumbed to virus-induced seizures (right panel). (B) Baseline weights of all mice were obtained, daily weights were calculated, and weight gain or loss was determined. Mice challenged with PBS (black triangles), MV alone (black circles), or LCMV alone (asterisks) showed modest increases in weight over the 34d observation period (>10 mice/group). The cohort of mice infected with both LCMV and MV were divided into two groups based on the severity of the illness that was observed: animals with mild illness (white diamonds) and with severe illness and mortality (white squares). Statistics were performed using the Wilcoxon Signed Rank Test. #: p = 0.0002; ##: p<0.0001.

Infection of mice with LCMV by an IP route generates a robust CD8^+^ T cell response that clears the virus within 7–10 dpi in the absence of overt disease. Similarly, MV infection of transgenic mice expressing a human measles receptor targeted to CNS neurons (NSE-CD46^+^ mice) results in activation of a protective adaptive response that resolves the infection in a similar timeframe, and without concomitant illness. Because these infections are tissue-restricted (LCMV to peripheral tissues and MV to the CNS), we explored the parameters of viral clearance, immune cell trafficking, and pathogenesis in mice infected simultaneously with both pathogens (**Figure 1A**).

NSE-CD46^+^ mice were challenged concurrently with MV by an IC route (1×10^4^ PFU) and LCMV by an IP route (2×10^5^ PFU) and monitored daily for weight loss and overt signs of illness. As shown in **Figure 1B** and **Table 1**, no weight loss was observed in any of the singly infected mice or uninfected controls over a 34d observation period and all mice appeared healthy throughout this timecourse. In contrast, approximately half of the mice challenged with both LCMV and MV showed dramatic weight loss (**Figure 1B**, white squares; **Table 1**, group 3), leading to death by 8–10 dpi. Within this group, all animals showed overt signs of CNS disease, including ataxia, tremors, and a hunched (kyphotic) and ruffled appearance. In the terminal stages of disease, mice developed severe seizures lasting >10s per episode; death was always coincident with these seizures. The co-infected mice that did not develop severe disease showed little change in weight (**Figure 1B** **,** white diamonds), but most did show moderate illness, including slight ataxia. Of note, most of the severely sick mice began to show signs of weight loss and illness within 3–4 dpi, whereas the mice that would go on to have mild disease and survive appeared asymptomatic at this timepoint.

**Table 1:** Morbidity following viral challenge.

Group	MV dose (PFU)*	LCMV dose (PFU)**	Interval (d)***	n	% morbidity****
1	1×10^4^	none	n/a	20	0
2	none	2×10^5^	n/a	20	0
3	1×10^4^	2×10^5^	n/a	68	44
4	1×10^3^	2×10^4^	n/a	17	35
5	1×10^4^ (UV)	2×10^5^	n/a	11	0
6	1×10^4^	2×10^5^ (UV)	n/a	13	23
7	1×10^4^	2×10^5^	7	16	0
8	1×10^4^	2×10^5^	30	12	0

*Mice were inoculated with the specified dose via intracerebral inoculation in a volume of 30 ul. For group 5, virus was inactivated by exposure for 15 min to a UV light source and absence of infectious virus verified by plaque assay.

**Mice were inoculated with the specified dose via intraperitoneal inoculation in a volume of 200 ul.

***For interval studies, mice were infected with MV, rested for the indicated number of days, and then infected with LCMV.

****Morbidity was determined by either weight loss greater than 20% and/or seizures, ataxia or persistent tremors.

To address whether the pathogenesis resulting from co-infection was attributable to increased viral load, we reduced the dosages of both MV and LCMV by 10-fold; however, identical results were obtained (**Table 1**, group 4). Moreover, when UV-inactivated MV was administered instead of replication-competent virus, no disease or weight loss was observed (**Table 1**, group 5). Interestingly, however, challenge of mice with UV-inactivated LCMV did result in morbidity in approximately 25% of infected mice, indicating that replicating LCMV was not required to elicit the pathogenic phenotype (**Table 1**, group 6). Finally, we found that the timing of the infections was critical: if the infections were separated temporally (7d and 30d tested, in which MV was given first, followed by LCMV), no pathogenesis was observed (**Table 1**, groups 7 and 8). Collectively, these data indicate that concurrent challenge of mice with two viruses triggers a novel pathogenic outcome that is not observed with either virus alone.

### Illness in co-infected mice is not attributable to mouse characteristics or to changes in viral tropism

To determine the basis of fatal neurological disease in some co-infected mice and to define the factors that govern the differential pathogenic response, we first considered possible differences in the infected mice themselves. Consequently, we evaluated whether sex or age distinguished mice that developed severe CNS disease from those that survived the milder disease. Mice of both sexes between 3–6 months of age were co-infected, and neither age nor sex correlated with pathogenic outcome (data not shown).

**Figure 2:**
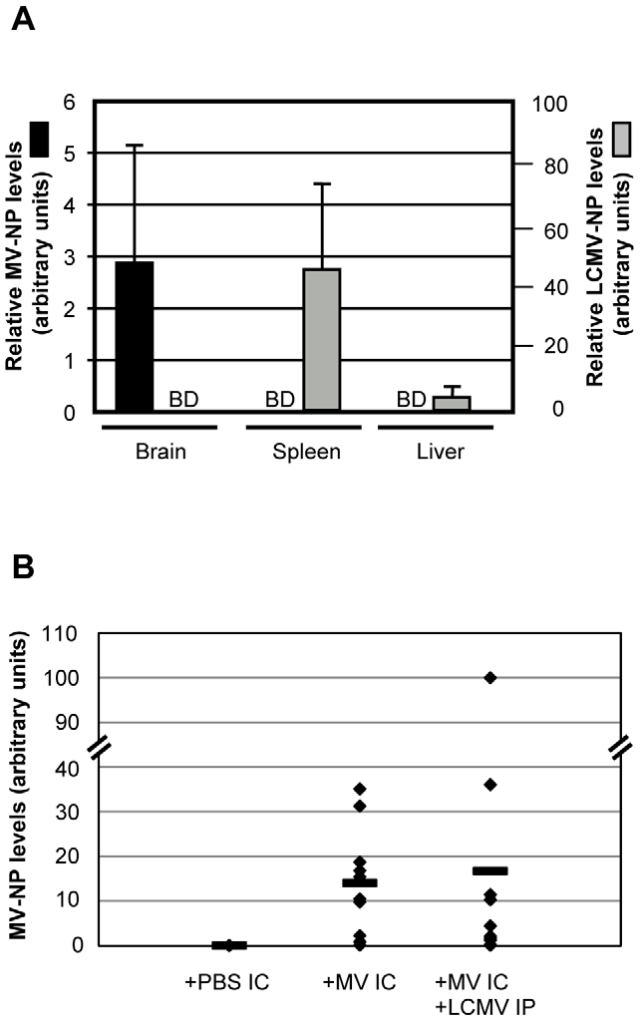
Tissue distribution of LCMV and MV in co-infected mice. (A) RNA was purified from the indicated tissues of co-infected mice at 4 dpi and amplified by quantitative RT-PCR for MV (black bars, scale on left) or LCMV (gray bars, scale on right). BD  =  below detection. (B) The extent of MV load was assessed in brains of mice infected with MV alone or with both MV and LCMV at 7 dpi. Data points represent individual mice, with averages shown as horizontal lines.

We next asked if co-infection altered MV or LCMV biology with respect to titer, tropism, or rates of viral clearance. As shown in **Figure 2A**, which depicts quantitative RT-PCR results from co-infected mice at 4 dpi, MV RNA remained restricted to the CNS and LCMV RNA to peripheral tissues, including the spleen and liver. Likewise, no evidence of LCMV immunopositive cells was found by immunohistochemistry in brains of co-infected mice (data not shown). When mice were evaluated at 2, 7, 12 and 30 dpi, though viral loads differed as these viruses were being cleared, the same tissue restriction was observed (data not shown). Moreover, the viral load of MV in the CNS was not appreciably different between singly and co-infected mice (**Figure 2B**). RNase protection assays (TNFα, TNFβ, CCL2, CCL3, CCL4, CCL5, CXCL2, CXCL10, CXCR3, IFNβ, IFNγ, IFNγR, VCAM1, IL-4, IL-12, IL-15, IL-18, iNOS) were performed and no significant change, in either levels or profile, was observed between MV infection alone and co-infection or between symptomatic and asymptomatic co-infected mice (data not shown). These data indicate that key aspects of the viral life cycle for both MV and LCMV, including tissue tropism and viral load, were not changed upon co-infection.

### Massive infiltration of CD8^+^ T cells into brains of co-infected mice

**Figure 3:**
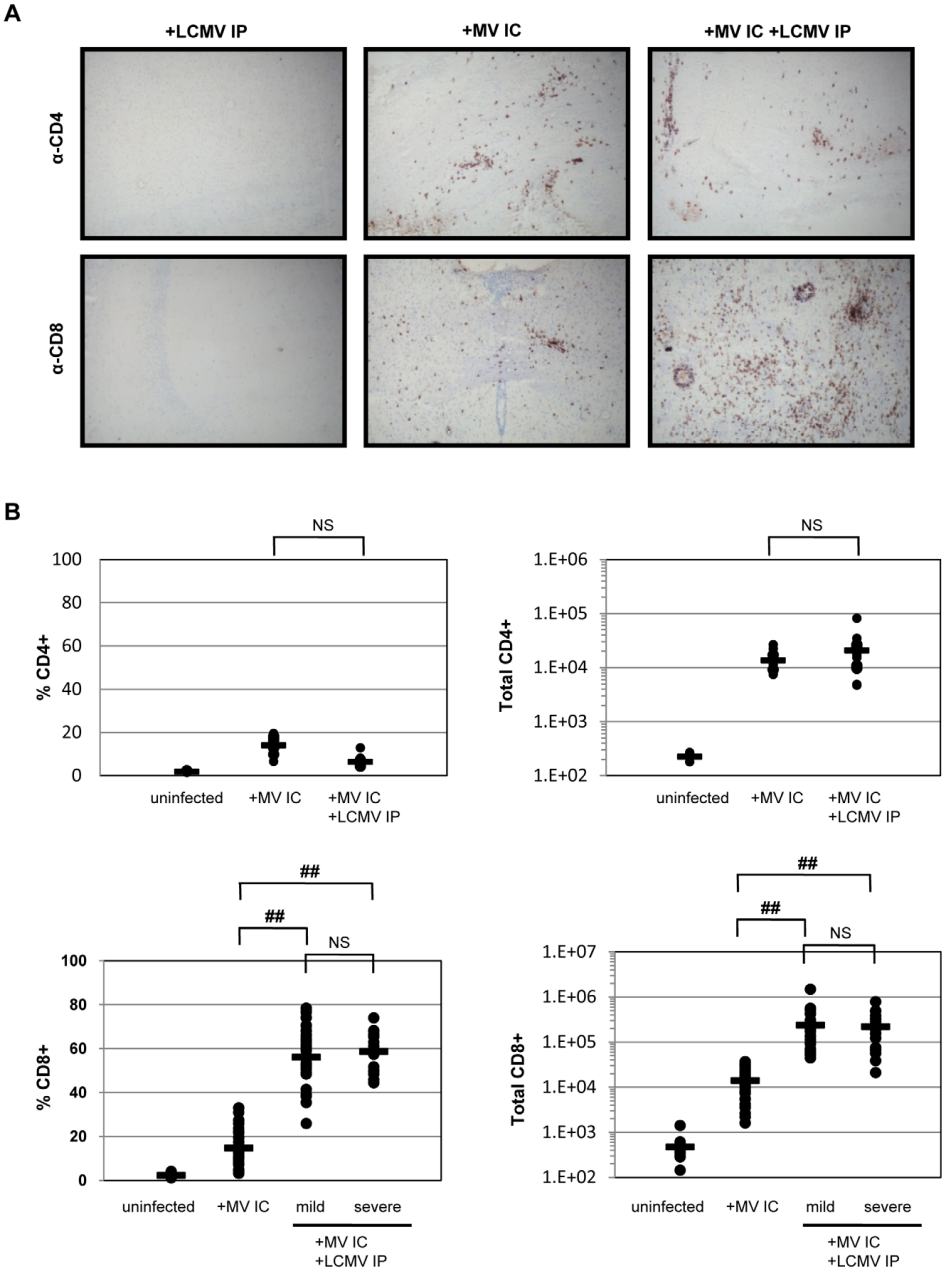
Immune response within the brains of co-infected mice. (A) Brains of mice infected with LCMV IP alone (left panels), MV IC alone (center panels), or both viruses (right panels) were harvested 7 dpi and serial sections were immunostained for either CD4^+^ T cells (top panels) or CD8^+^ T cells (bottom panels) using DAB (red/brown staining) and counterstained with hematoxylin (gray/light blue staining). Original magnification = 100X. (B) Brains were removed from the indicated mice at 7 dpi and homogenized. Mononuclear cells were extracted and subjected to staining using fluorophore-conjugated antibodies, followed by flow cytometry. Using this flow cytometry data, together with cell counts obtained using a hemocytometer, the total number of CD4^+^ and CD8^+^ T cells, as well as the percent of total CNS infiltrate, was calculated. Values for individual mice are shown, with averages shown as horizontal bars. Statistics were performed using the Wilcoxon Signed Rank test. ##: p<0.0001; NS: not significant.

We next evaluated the host immune response within brains of infected mice, first using standard immunohistochemistry for the presence of both CD4^+^ and CD8^+^ T cells in horizontal brain sections, followed by *ex vivo* isolation of brain-infiltrating immune cells combined with flow cytometry and hemocytometer counts. Representative horizontal plane images are shown in **Figure 3A**. As published previously [9], [17], NSE-CD46^+^ transgenic mice infected with MV IC show substantial, and approximately equivalent, infiltration of both CD4^+^ and CD8^+^ T cells at 7 dpi (**Figure 3A**, center panels). As expected, T cells typically accumulate in regions where MV antigen is prevalent (data not shown). Brains from LCMV-infected mice had no evidence of T cell infiltration into the CNS (**Figure 3A**, left panels), because the CNS is not a target organ for infection by LCMV via the IP route (**Figure 2A**). When mice were challenged with both LCMV and MV and examined histologically at 7 dpi (**Figure 3A**, right panels), the number and proportion of CD8^+^ T cells in the CNS was steeply elevated compared to singly infected mice (**Figure 3A, 3B**), increasing from approximately 14,000 cells in MV-infected brains to 170,000 cells in brains of mice challenged with both viruses. This represents a 12-fold increase over the single MV infection and a >250-fold increase compared to uninfected mice. As shown in the bottom right panel of **Figure 3A**, CD8^+^ T cells were found distributed throughout the brain, though somewhat concentrated in patches that correlated with sites of MV replication (data not shown), as well as surrounding vascular structures, ventricles and meninges. While there was a range in the number of lymphocytes isolated from individual brains, the increase in CD8^+^ T cells was observed in all co-infected mice that were tested, regardless of illness (**Figure 3B**). In contrast, the number of CD4^+^ T cells was only slightly increased in co-challenged mice, compared to levels seen in MV-infected mice alone (avg. 13,500 vs. 22,000; **Figure 3A, 3B**). Similarly, flow cytometry revealed no significant increases in other immune cell populations (B cells, NK cells, NKT cells, macrophages, neutrophils, dendritic cells; data not shown). Thus, the lethal CNS disease that occurs in approximately half of the co-infected mice is not simply attributable to the abundance of infiltrating cells within the brain parenchyma, and appears to involve CD8^+^ T cells.

### LCMV-specific CD8^+^ T cells are found in the brains of co-infected mice

**Figure 4:**
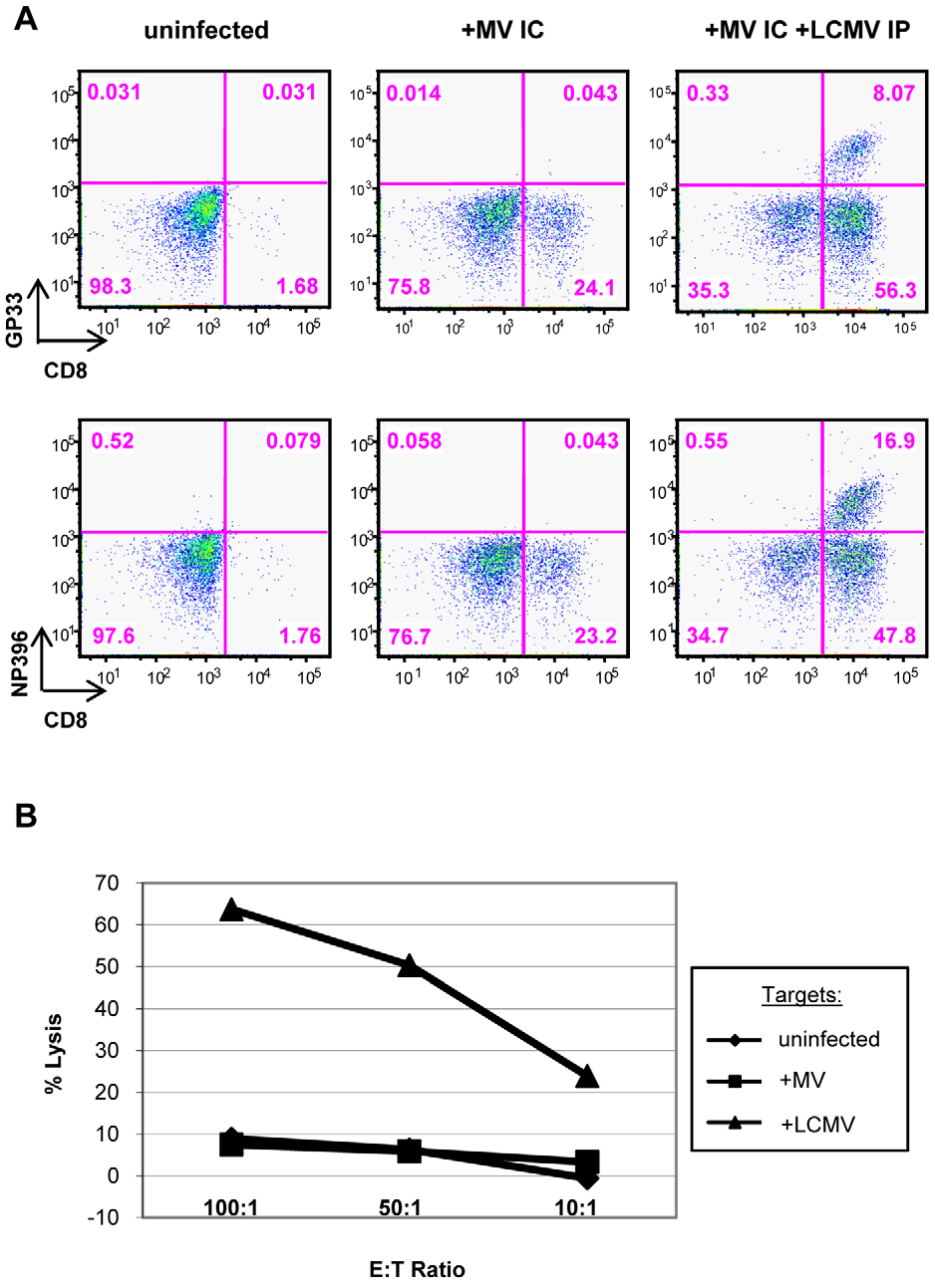
LCMV-specific CD8^+^ T cells are found within the brains of co-infected mice. (A) T cells were isolated from brains of mice that were either uninfected (left panels), infected with MV IC alone (center panels), or infected with both LCMV IP and MV IC (right panels), as described in Figure 3. Cells were dual stained for CD8 (x-axis) and for one of two immunodominant LCMV epitopes (y-axis), using specific tetramers (GP33: top panels; NP396: bottom panels). (B) A standard ^51^Cr release assay was performed by incubating effector splenocytes from LCMV-infected mice (6 dpi) with uninfected (diamonds), MV-infected (squares), or LCMV-infected (triangles) target cells at the indicated ratios. Released ^51^Cr was measured in a gamma counter and specific release was calculated.

LCMV potently activates CD8^+^ T cells; thus, we used LCMV-specific tetramers to determine the proportion of CNS-infiltrating CD8^+^ T cells of LCMV-specificity. Tetramers specific for the two major LCMV epitopes on the H-2^b^ background (GP33 and NP396) were employed (provided by Dr. John Wherry). As shown in the representative data in **Figure 4A**, right panels, 8% (mean: 6.1 ± 2.0%) and 17% (mean: 15.2 ± 3.8%) of the lymphocytes extracted from co-infected brains were CD8^+^ T cells specific for LCMV GP33 and NP396, respectively. Based on these two immunodominant peptides, >35% (37.6 ± 5.8%) of the total infiltrating CD8^+^ T cells (>21% of the total brain-infiltrating immune cell population) were specific for LCMV within the brains of co-infected mice. This is likely a substantial underestimate, as this does not account for T cells specific for minor LCMV epitopes. LCMV tetramers did not label any lymphocytes isolated from brains of mice infected with MV alone, indicating that there is little to no cross-reactivity between LCMV and MV, as measured by this assay. This was further supported in standard CTL assays, in which splenocytes isolated from mice infected with LCMV IP did not recognize MV-infected target cells (**Figure 4B**).

### The manifestation of CNS disease in co-infected mice is identical to that seen in mice challenged with LCMV by an intracerebral route

**Figure 5:**
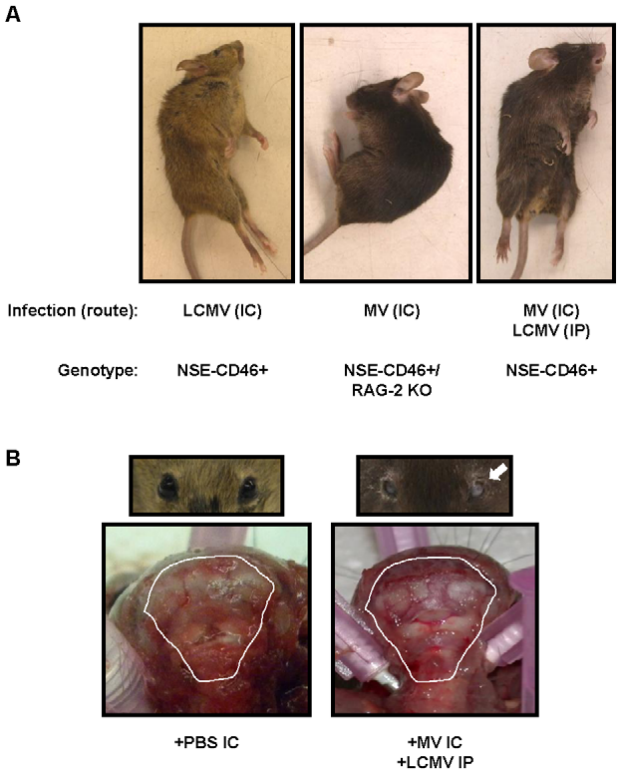
Postmortem examination of neuropathogenesis in co-infected mice shows signs of brain herniation. (A) Representative mice infected with LCMV IC (left panel), MV IC (in NSE-CD46^+^ RAG-2 KO mice) (center panel), and both LCMV and MV (right panel) are shown exhibiting characteristic, post-mortem postures. (B) The scalp was removed from moribund co-infected mice or PBS treated controls to expose the skull; photographs were taken of the rear of the brain and cervical spinal cord. Note compression of white matter (cerebellum) against the posterior skull in co-infected animals. Mice were also examined for unilateral pupillary dilation (mydriasis) (seen here in mouse's left eye; white arrow). Photographs are representative of 5 to 10 mice per group.

Interestingly, the posture of mice that die following co-infection is identical to that seen in mice challenged with LCMV by an IC route (**Figure 5A**). IC LCMV infection of immunocompetent mice with as few as 1 PFU results in overt signs of illness by ∼6 dpi, including ruffled fur, ataxia and tremors, which invariably lead to seizures and death by 6.5–7 dpi. The decerebrate posture following LCMV death is unique to LCMV, as mice that succumb to other CNS infections (such as RAG-2 KO mice following MV infection; **Figure 5A**, second panel) or mice infected with poliovirus (data not shown), do not develop seizures or a similar post-mortem posture.

Recently, we showed that death following LCMV IC challenge coincided with apparent compression of the brain against the skull [14]. Moreover, all mice dying of LCMV by the IC route had unilateral pupillary dilation (mydriasis), which is likely attributable to uncal herniation of brain tissue through the foramen magnum. Similar observations were found in all moribund mice that were infected with both LCMV IP and MV IC (**Figure 5B**, right panels), implying a common mechanism of disease.

It is well established that mortality following IC challenge with LCMV is mediated by CD8^+^ T cells [18], [19]. Thus, given the massive infiltration of CD8^+^ T cells into the brains of co-infected animals, combined with the observed similarities in gross pathology between the co-infected mice that die and those challenged with LCMV IC, we next elucidated the role that CD8^+^ T cells play in the neuropathogenesis observed in the co-infected animals.

### CD8-depleted mice exhibit delayed pathogenesis

**Figure 6:**
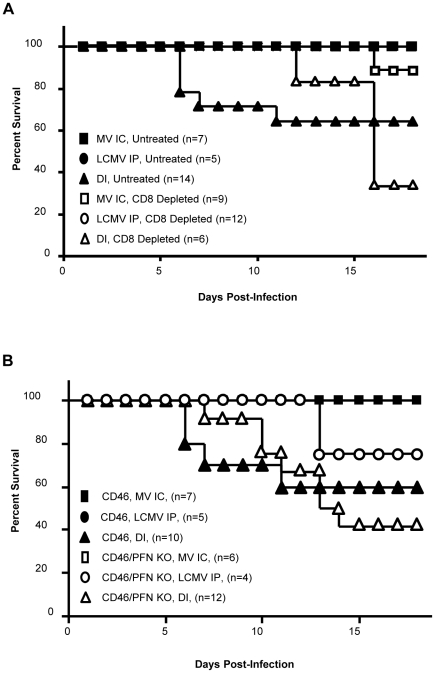
Pathogenesis in co-infected mice is CD8^+^ T cell dependent and perforin independent. (A) NSE-CD46^+^ mice were injected IP with 150 µg of CD8-depleting antibody (clone 2.43), or an isotype control antibody, and challenged with MV IC alone (closed/open squares), LCMV IP alone (closed/open circles), or both MV IC and LCMV IP (closed/open triangles). Mice were monitored daily for survival. (B) Mice of the indicated genotypes were challenged with MV IC alone (closed/open squares), LCMV IP alone (closed/open circles), or both MV IC and LCMV IP (closed/open triangles) and monitored daily for survival.

CD8-depleted mice were challenged with both MV IC and LCMV IP (**Figure 6A**). As expected, CD8^+^ T cell depletion delayed the onset of pathogenesis by approximately 6d, indicating that CD8^+^ T cells are important for the mechanism of pathogenesis. While some co-infected CD8-depleted mice did succumb at later timepoints, they displayed symptoms similar to MV IC infection of NSE-CD46^+^/RAG-2 KO mice (hunched/kyphotic posture and ruffled appearance) and not those observed with symptomatic co-infected immunocompetent mice (seizures leading to death and the characteristic decerebrate posture). Furthermore, a proportion of CD8-depleted animals challenged with MV IC alone also succumbed with MV-like symptoms, confirming that, in the absence of CD8^+^ T cells, mice are susceptible to an intracerebral MV infection.

To elucidate the mechanism of pathogenesis mediated by CD8^+^ T cells in the co-infection model, we infected mice which lack the pore-forming protein perforin, which is required by cytotoxic lymphocytes to mediate granzyme-driven lysis of target cells. Co-infected NSE-CD46^+^/PFN KO mice succumbed in a similar fashion to NSE-CD46^+^ immunocompetent mice, both temporally and symptomatically (**Figure 6B**). This indicates that perforin does not a play a central role in CD8^+^ T cell-mediated pathogenesis in co-infected mice. At later time points (>15 dpi), NSE-CD46^+^/PFN KO animals succumbed somewhat more rapidly than NSE-CD46^+^ immunocompetent mice; however, they did so with symptoms identical to those seen during MV infection of immunocompromised NSE-CD46^+^/RAG-2 KO mice [9]. This decline may be attributable to the observation that PFN KO animals succumb to a LCMV IP alone challenge at later timepoints, whereas immunocompetent mice survive such a challenge.

### Edema correlates with morbidity in co-infected mice

In LCMV IC-challenged mice, brain herniation results from an increase in intracranial pressure, which causes the brain tissue to shift from an area of higher pressure to one of lower pressure. To determine whether the co-infected mice were dying from brain herniation (as suggested by their postmortem posture in a CD8-dependent fashion), we examined various mechanisms that could result in increased intracranial pressure.

**Figure 7:**
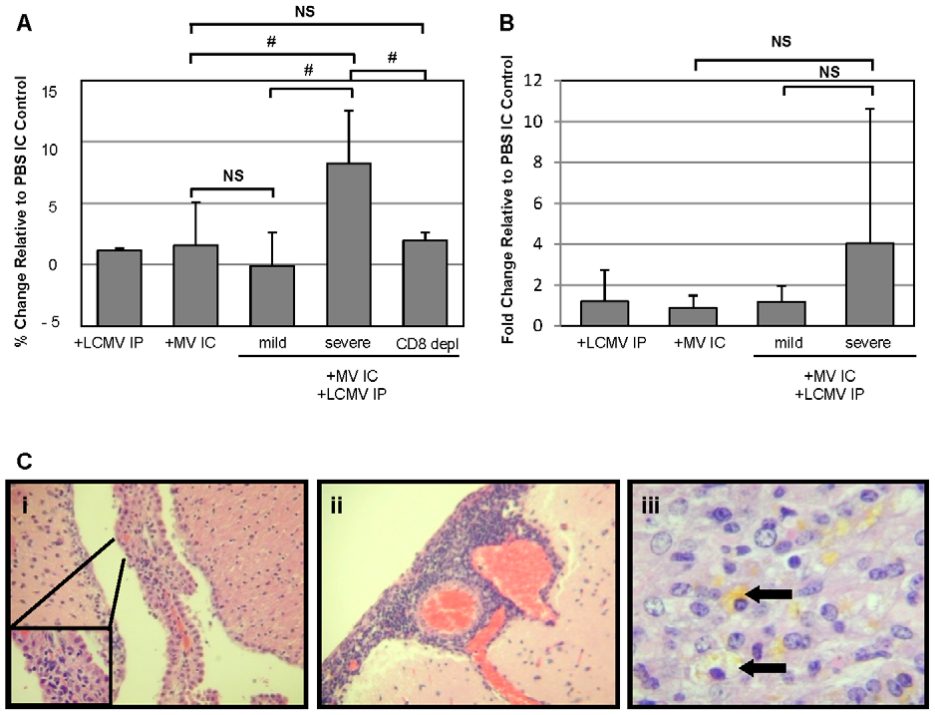
Edema, but not blood brain barrier, changes correlate with pathogenesis in co-infected mice and is dependent on CD8^+^ T cells. (A) Brains were removed 7 dpi from mice infected as indicated and weighed before and after dehydration in a vacuum oven. Total water weight was determined for each mouse and normalized to values from PBS IC mice; averages are shown as grey bars, with standard deviation indicated. Statistics were performed using the Wilcoxon Signed Rank test. #: p = 0.03; NS: not significant. (B) Mice, infected and treated as indicated, were anesthetized at 7 dpi and injected with Evan's Blue, followed by saline perfusion. Total Evan's Blue that diffused into the brain was determined by spectrophotometry following brain homogenization and clarification. Data were normalized to values from mice inoculated with PBS IC, and averages are shown as gray bars, with standard deviation indicated. Statistics were performed using the Wilcoxon Signed Rank test. NS: not significant. (C) Representative photomicrographs were taken from brain sections of mice with severe disease following co-infection with LCMV IP and MV IC. Evidence of choroid plexus inflammation (i), meningitis (ii), and hemosiderin deposition, indicative of capillary bleeding (iii), are shown. Original magnifications: A, B  =  640X; C  =  800X.

Edema increases intracranial pressure, which can then lead to herniation. To directly ascertain if moribund mice had evidence of cerebral edema (elevated brain water weight), brains were removed at 7 dpi from: i) control PBS IC mice, ii) mice that were challenged with LCMV by an IP route (not expected to lead to CNS water volume changes), iii) MV IC (which results in immune cell infiltration, but no CNS disease), or iv) mice infected with both viruses. For the co-infected group, mice were further separated into those with severe (fatal) illness and those with the milder, nonfatal disease. Mice challenged singly with either LCMV IP or MV IC had water volume values that were not appreciably changed from uninfected or PBS-challenged mice (**Figure 7A**). (Water weight raw values ranged from 72–81%). Similarly, co-infected mice with mild disease showed no substantial edema (average of 77%, and a range of 73–79%). In sharp contrast, severely sick, co-infected mice showed a statistically significant increase in water volume (>7% over baseline), with an average of 83%, ranging from 80–87%, strongly suggesting that edema is mechanistically linked to mortality in this model system. As further evidence of a role for CD8+ T cells in this process, CD8-depleted mice did not show a significant increase in edema.

We also evaluated the integrity of the blood-brain barrier (BBB), as determined by extravasation of albumin-bound Evan's Blue into the brains of infected mice, as a possible cause of increased brain edema and resulting herniation. As shown, all singly infected mice and co-infected mice with mild symptoms showed negligible increases in Evan's Blue levels, indicative of an intact barrier, at 7 dpi (**Figure 7B**). Severely sick, co-infected mice showed only modest BBB permeability. Though there was variability in the values within the severely sick group, with some mice exhibiting high levels of Evan's Blue, there was no statistical significance in the overall increase in permeability. This is consistent with the LCMV IC model: while the blood-brain barrier is permeabilized in many of these mice, the changes do not correlate with the onset of disease.

Finally, we assessed the histopathology in brains of moribund mice to determine potential causes for the observed cerebral edema (**Figure 7C**). In all animals evaluated, inflammation within the choroid plexus (i) and meninges (ii) was found, along with evidence of parenchymal damage, as determined by the extensive deposition of hemosiderin within the parenchyma (iii, arrows), indicative of capillary bleeding. Interestingly, the moribund co-infected animals had greater levels of meningitis than the animals which survived challenge with both viruses (data not shown).

Collectively, these data suggest that the processes that mediate pathogenesis in co-infected NSE-CD46^+^ mice are similar to those observed in mice challenged with LCMV by the lethal IC route. However, unlike the LCMV IC infection in which all mice succumb, co-infection results in mortality in only approximately 50% of infected individuals. Specifically, disease is CD8-dependent, yet perforin-independent, and correlates with increased cerebral edema and evidence of both meningitis and encephalitis.

## Discussion

This paper describes the development of a mouse model of tissue-restricted, simultaneous viral challenges: MV is restricted to CNS neurons due to transgenic specification of the receptor, and LCMV is limited to peripheral tissues due to the route of inoculation. While single infection with either virus alone results in immune-mediated protection in the absence of disease, co-infection results in a high degree of morbidity and mortality, apparently triggered by migration of LCMV-specific CD8^+^ T lymphocytes into the MV-infected CNS.

The pathogenesis observed in moribund co-infected mice is indistinguishable from that seen in mice challenged with LCMV by an IC route. We previously showed that disease in LCMV IC-infected mice is attributable to increased brain water weight (edema) and reactive periventricular disease, culminating in physical manifestations consistent with uncal herniation (seizures, decerebrate posture, mydriasis) [14]. In the present study, similar increases in edema were observed in moribund, co-infected mice. While changes in blood-brain barrier integrity were also noted in these mice (as also noted in LCMV IC infected mice), increased permeability was neither significant nor consistently correlated with the timing of disease. Therefore, though blood-brain barrier damage may contribute to neuropathogenesis, it does not appear to be the precipitating cause of mortality in our mouse model. Importantly, active infection of the CNS was a prerequisite for disease, as instillation of the CNS with UV-inactivated MV did not result in either elevated T cell recruitment or neuropathogenesis. Thus, the acute CNS infection (which is pathogenically inert when delivered alone) likely induces recruitment signals, including chemokines [20], and adhesion molecules on barrier endothelium, that then aid lymphocyte recruitment. Of relevance, the receptor for CXCL10, CXCR3, is expressed by a majority of activated, LCMV-specific CD8^+^ T cells, which would presumably allow them to traffic toward the MV-initiated recruitment signal [21], [22]. These chemoattractant signals would be expected to recruit all activated immune cells, regardless of specificity. While other models have shown that lymphocytes can be recruited to tissues that do not express cognate antigen [4], [23], [24], to our knowledge, the MV/LCMV co-infection model is the first to show that this phenomenon can have pathogenic consequences.

While the parallels between mortality following co-infection and the classic LCMV IC infection allowed us to define neuroanatomical events that were consistent with mortality, the precise mechanism by which LCMV-specific T cells trigger damage within the CNS remains unknown. It does not seem likely that the massive infiltration of T cells into the brain can, alone, account for disease, as there was no direct correlation between total lymphocyte counts in the CNS and disease severity. Nevertheless, the possibility that migration of T cells to a particular site within the brain may contribute to enhanced vulnerability cannot be excluded. Using immunodepleted and immune knockout mice, it is clear that the neuropathogenesis following co-infection in this model is dependent on CD8^+^ T cells, but independent of their effector function involving perforin.

A key question that is the basis of our ongoing work is to define what LCMV-specific T cells “see” to trigger disease, since we detected no cross-reactivity between MV and LCMV, and by multiple methods, no LCMV RNA or proteins were detected in the CNS at any timepoint tested, from 2–30 dpi. Moreover, co-infection with UV-LCMV IP together with MV IC triggered neuropathogenesis, indicating that replicating LCMV need not be present in the CNS. Classically, the expression of effector functions by CD8^+^ T cells is reliant on the recognition of antigen. However, together with IFN-γ, the innate cytokines, IL-12, IL-18, IL-23 and IL-27, have all been shown to be potent activators of T cells that act in an antigen-independent manner, as well as independent of TCR ligation and signaling [25], [26]. Binding of these interleukins to their respective receptors on T cells alone causes these CD8^+^ T cells to become activated and release IFN-γ and TNF-α. These early functions of CD8^+^ T cells are important components in the host response to such pathogens as influenza A virus, *Lysteria monocytogenes*, *Cryptosporidium parvum*, and tumors [26]–[30]. Together, these data suggest that CD8^+^ T cells may indeed play a role in immunopathogenesis if they have been independently activated and then ‘misrecruited’ to another, independent site of infection.

Neither changes in tropism nor changes in the extent of infection of either virus were seen to change following co-infection. While our PCR-based and IHC-based studies found no evidence of LCMV infection within the brain, work by others suggests that brain endothelial cells and epiplexus cells can present antigen in the absence of direct infection, similar to cross-presentation by antigen presenting cells [31]–[33]. Thus, it is possible that in these co-infected animals, brain endothelial cells or epiplexus cells present LCMV epitopes picked up from the CSF, in the absence of direct infection. Evaluation of this mechanism as a possible cause of neuropathogenesis is an ongoing effort, and adoptive transfer studies using activated LCMV-specific CD8^+^ T cells into a MV-infected recipient are underway. However, we are cautious about a result of a lack of neuropathogenesis in these experiments, as a “primed” microenvironment within the periphery may be an important component in activation of such cells.

What general implications can be inferred from these results? Mouse models have been essential for the study of viral pathogenesis, both in aiding our understanding of the viral life cycle in vivo and in revealing critical aspects of immune response induction, recruitment, and function. Nevertheless, the "single-challenge" approach that most viral pathogenesis studies employ--in which immunologically naïve adult animals are inoculated with only one pathogen and evaluated thereafter--does not approximate the complexity of immune history or host responses to concurrent immune challenges that occur in humans.

A prominent example of coinfection with enhanced pathology is human immunodeficiency virus (HIV) and hepatitis C virus. For pathogen combinations such as this, the mechanism of increased pathology is well understood: Pathogen A causes immunosuppression of the infected host, thereby setting up a permissive environment for Pathogen B to establish an unrestricted infection [34]. The issue of immunosuppression is one that we have not explored, even though it is known that MV reduces responses to a co-infecting agent (i.e. HIV) [35]. At this point, we do not believe that this is a major confounder for our experiments since all arms of the immune response examined in our model are either increased or remained at baseline, when compared to either single infection alone. For other diseases, such as Burkitt's lymphoma, polymicrobial infections (Epstein-Barr virus (EBV) and Plasmodium falciparum malaria) are clearly associated with the disease outcome, but the causative mechanism is still elusive [36]. Thus, polymicrobial infections – especially in developing regions of the world – are common, though how these pathogens and their respective immune responses interact to result in pathogenic outcomes remains unknown.

While making direct connections between this model system and human diseases would be premature, a similar phenomenon could account for both the reported increase in inflammatory cells found in the brain of patients presenting with MS and ALS [37]–[40], as well as the lack of consistent and convincing presence of any specific pathogen in the brains of affected individuals [41]–[44]. A large number of CNS diseases of unknown etiology have been proposed to have viral triggers [45]–[48], though no single pathogenic agent has been linked with any chronic CNS disease, perhaps because it is generally assumed that the viral trigger must be co-localized with the observed damage. Our co-infection model of immune cell recruitment suggests that this may not need to be the case – the virus infection and damage sites may be spatially distant, provided that a recruiting signal (e.g., a chemokine gradient) is present within the CNS.

While our studies focused on viral pathogens, the results can be considered more broadly in terms of any agent or situation that can trigger an immune response. For example, concurrent immune responses to other antigens (e.g., tumors, allergens, autoantigens) might also contribute to pathology resulting from infection-irrelevant immune effector trafficking. In summary, while single-pathogen mouse models will continue to be powerful tools to explore the induction and function of immune cells and their mediators, more complex animal models, which consider both pathogenic and non-pathogenic triggers of host immunity, will be required to fully reveal the diversity of ways by which immune-mediated neurological diseases occur.
